# “Tirer d’eux leurs secrets”: Leibniz on Artisanal Knowledge and “Secret” Geometry

**DOI:** 10.1007/s00048-025-00414-8

**Published:** 2025-06-06

**Authors:** Yoav Beirach, Michael Friedman

**Affiliations:** 1 Research Group Experience in Pre-Modern Sciences, Max Planck Institute for the History of Science, Boltzmannstraße 18, 14195 Berlin, Germany; 2 Hausdorff School for Mathematics, University of Bonn, Endenicher Allee 60, Bonn, Germany

**Keywords:** Gottfried Wilhelm Leibniz, Artisanal knowledge, Mechanical and transcendental curves, Horology, Weaving and textile practices, Secret geometry, Christiaan Huygens

## Abstract

What was Leibniz’s approach to artisanal knowledge? And how did he consider it with respect to mathematical, and more concretely, to geometrical knowledge? On the one hand, Leibniz emphasizes several times in his writings that one should extract “secrets and inventions” from the artisans. On the other hand, Leibniz points out that such artisans cannot formulate by themselves the geometric principles at the base of their machines. In this paper, we examine these intricate relations between Leibniz’s reflections on artisans, especially clockmakers and textile workers, as well as his thoughts on mechanical and geometric knowledge. Leibniz’s considerations of various artisanal machines, like clocks and looms, lead us to discuss his wish to expand geometry, presenting these machines as embodying a “secret,” “hidden,” or even “deeper” or “more profound” geometry.

## 1. Introduction

In a letter from May 1673 to Christian Habbeus, the young Gottfried Wilhelm Leibniz describes his fascination with the artisans of Paris he had met a year earlier, as well as with artisanal knowledge in general. Along with other artisans, he mentions textile workers who operate a special kind of textile machine—the stocking frame (a machine that mechanized knitting)—and clockmakers. Moreover, he repeatedly notes that one should “draw” (that is, extract) “secrets and inventions” from these artisans.^1^ First he mentions that the king of France convened all artisans to extract such inventions; then he notes that generally “it would be important to fish [*pécher*] from the workers here the fine and delicate details of their secrets”;^2^ and later he underlines that “for my part, I had the opportunity not only to frequent a good number of good artisans, but also to extract [*tirer*] something from them.”^3^ This fascination and admiration was not just a facet of Leibniz’s youth. During the fall of 1688, he prepared notes for a lecture he was to deliver in the presence of Kaiser Leopold I. The opening paragraph of these notes presents a list of important recent technological inventions. The first inventions listed come from the “*practica artium*, … whose uses are illuminating. Accordingly, I have many new inventions, some of which are my own, some of which are the work of others, but I have revealed them only to a few.”^4^ The first two machines mentioned are Leibniz’s own *machina arithmetica* and another called *machina deciphratoria*, both with obvious connections to intellectual practices and abstract reasoning.^5^ Besides these two, Leibniz notes, “There are people who have made such advancements with *machinis textoriis*, so that floral ribbons and cloths (textiles) can be produced with incredible convenience and speed, and little of this is made in Germany, but rather abroad,” referring later to *Bandmühlen* (ribbon looms) when again describing these *machina textoria*.^6^ The next invention mentioned is a clock: “I have thought up an invention of clock movements that go neither slower nor faster …”^7^ Leibniz considered the *machinis textoriis* to be roughly as important as his own calculating machines and his clock, suggesting that all these inventions may have had a similar status in his understanding of recent technological advancements.

Two questions hence arise: First, what was Leibniz’s relationship with artisanal knowledge and the artisanal practices of his time? Second, and more importantly, to what extent and how is this “secret” artisanal knowledge related to mathematics, as vaguely implied in the 1688 notes? The aim of this paper is to argue that such an association was not vague at all: the relationship between an appreciation of the secrets of artisanal practices and Leibniz’s view that such practices may be mathematized (or rest on a mathematical basis) and hence expand mathematics and especially geometry emerges in Leibniz’s writings of the 1670s and ’80s. Indeed, as we will show in this paper, Leibniz considered several artisanal machines—including Huygens’s pendulum clock and a number of textile machines—as acting according to the rules of mechanics or to the principles of a hidden geometry. According to Leibniz, this also points implicitly or explicitly to the fact that these machines work according to principles that show or embody mechanical curves (in the sense of René Descartes), or in Leibniz’s later term: transcendental curves. However, it is important to underline two issues: first, his use of the terms “algebraic” and “transcendental” does not necessarily correspond to their modern definitions, and second, Leibniz developed his own interpretation of these terms in the 1670s (Breger 1986; Probst 2012). But as we will see, Leibniz noted during that time and later that the curves along which the artisanal machines move should also be considered geometric.

Nevertheless, Leibniz’s attitude toward artisans was not mere fascination. First, he expresses different and changing views concerning the relationship between artisans, their practices, and their machines. As will be elaborated, with the example of clockmakers, Leibniz mainly considers the makers *of machines*, and the rules and principles (mechanical or geometric) according to which they worked (or should have worked). With textile practices, on the other hand, Leibniz considers the artisan a geometer only when they are able to *mechanize a practice*; in this case artisans are either the makers of *things* or the constructors of *machines*, two very different categories. It is mainly to the second category and the associated machines that Leibniz ascribes geometric knowledge.^8^ Second, in his 1684 “Meditationes de cognitione, veritate, et ideis,” Leibniz presents various distinctions with respect to types of knowledge. Knowledge can be obscure or clear, and clear knowledge can be confused or distinct.^9^ Regarding confused knowledge (or notions), Leibniz notes that painters and other artists (*artifices*) know immediately when a work of art is made well or badly, but when asked the reasons for these judgments they are unable to explain them.^10^ While one may argue that this claim refers only to works of art, it should also be noted that a similar view regarding artisanal knowledge is evident when in 1685 Leibniz copied Joachim Jungius’s manuscript entitled *Logica de notionibus*. In this copy, in a section called “De Notionibus Distinctis et Confusis” (On Clear and Confusing Notions), one finds a short discussion of Jungius on the *Strumpfband* (garter): a narrow band of fabric fastened around the leg to hold up stockings, being a “beautiful example of confused and distinct thought.”^11^ The manual activity of knotting and knitting the garter is referred to as embodying confused knowledge. However, this same activity may be turned into distinct knowledge once one coins specific terms.^12^ Such an analysis supports the claim of Matthew L. Jones: Leibniz, while appreciating artisanal knowledge, eventually considered such practices “decidedly inferior to theoretical knowledge as a way of knowing, however much artisanal skill surpassed theoretical knowledge in understanding the particulars of nature and the means to manipulate it” (Jones 2016: 82).

Leibniz’s ambivalent approach to artisanal knowledge was quite common in the early modern period. One can mention two representative examples: First, in the early seventeenth century, Francis Bacon not only aimed to transform craft knowledge into universal knowledge, he also called for nature to be “tortured” in order to extract knowledge from it, as if nature, as well as artisans, held secrets (Young 2017: 529).^13^ Second, in 1728, Christian Wolff stated that “the facts of nature are sometimes so hidden [*latent*] that they do not spontaneously present themselves to one who is attentive. … Hence it follows that philosophy would be helped if phenomena observed in the workshops of craftsmen [*officinis artificium*] and elsewhere in the arts [*arte*] … were collected and accurately described. For such things constitute a part of secret historical knowledge [*cognitionis historicae arcanae*] which cannot be obtained otherwise by the senses” (Wolff 1963: 12). Such intersections between natural philosophy and artisanal knowledge have been discussed extensively in the secondary literature from various points of view: as a trading zone (Long 2011), a legitimation or delegitimation of (artisanal) knowledge (Young 2017), an acknowledgment of various artisanal epistemologies (Smith 2022), and a recognition of the rise of mathematical practitioners (Cormack, Walton and Schuster 2017; Werrett 2019).^14^ In this paper, however, we argue that Leibniz’s approach to artisanal knowledge—an approach that echoes similar (though certainly not identical) views concerning the French *artiste*—was based not on a symmetrical trading zone or mere legitimation, but rather a distanced, careful, and fascinated examination.^15^ Even if this approach shares many characteristics with other thinkers of his time, as well as with historical accounts of the relationship between artisans and natural philosophers in the seventeenth century, Leibniz exhibits a unique, even idiosyncratic, relationship with artisanal knowledge. This unique relationship can be seen in the way Leibniz interweaves his thoughts on geometric and transcendental curves, “secret geometry,” and artisanal knowledge. While his claim that the artisan’s knowledge is secretive is probably also an expression of the fact that the artisan, unlike most people, had a unique access either to nature—that is to materials found in nature—or machines, Leibniz viewed these practices and machines as also embodying hidden geometric knowledge that should be extracted.

Section 2 presents Leibniz’s intricate reflections on artisans, especially clockmakers and textile workers, with a discussion on Leibniz, clockmaking, and clockmakers. In Leibniz’s writings on artisanal knowledge, a comparison between his and Huygens’s clocks gives rise to a distinction between mechanical and geometric knowledge, the former expected by Leibniz of artisans. In Section 3, we move on to discuss Leibniz’s views on textile artisans and textile machines; here too, the question arises whether the various machines are based on geometric or mechanical principles. Our considerations of these machines lead us to discuss Leibniz’s wish to expand geometry, presenting these machines as embodying a secret, hidden, or even deeper or more profound geometry. The broader context of this wish is discussed in Section 4, linking it to the early-eighteenth-century figure of the “geometric genius” and *artiste*—an artisan who exhibited increased interest in the mechanical arts—as well as the question of the limits of geometric knowledge in the late seventeenth century.

## 2. “Rarely Does the Mind Rise to a Different Principle of Discovery”: Leibniz and Clockmaking

Leibniz had numerous interactions, including long-term relationships, with a number of clockmakers and clock inventors throughout Europe. He visited clockmakers’ workshops,^16^ publicly commented on the quality of their work,^17^ collected and kept the publications of a contemporary master,^18^ directed the work of clockmakers on his computing machine (Jones 2016: 56–87), and took an active part in legal disputes on the priority and patenting of timekeeping devices.^19^ He also practiced the art of clockmaking himself and claimed to have had a great interest in clockmaking since at least 1671. He invented a spring-based timekeeping mechanism and in 1675 published a detailed article on the mechanics of this device,^20^ as well as (posthumously) a more general piece on clockmaking and the art of horology.^21^ In his correspondence, personal notes, and published works, Leibniz often repeated that he had discovered a “purely mechanical” principle of timekeeping as early as 1671. All of this shows that Leibniz had a close relationship to clockmaking. While the clock was among the central metaphors of the early modern period (for Leibniz as well), the short review presented above demonstrates that he was also intimately engaged with the material and practical dimensions of clockmaking.^22^

Leibniz was already working on his own clock in the early 1670s. A full description of the invention was published in 1675 in the *Journal des sçavans*. Shortly after its publication, Leibniz sent a copy to Henry Oldenburg accompanied by a letter. In an unsent draft of this letter, Leibniz emphasizes how his device utilizes a new principle to achieve equality of periods: “I am therefore sending you, as you see, a description of the principle of equality in the clock from my future invention, which has nothing in common with the isochronism of vibrating pendulums or other elastic things, which, however, have been used by all until now.”^23^ This principle “used by all” refers to the principle of the isochronism of the cycloid pendulum described by the Dutch mathematician, natural philosopher, and inventor Christiaan Huygens in his 1673 *Horologium Oscillatorium.* Leibniz, who was his student in Paris and later corresponded with him extensively, also mentions Huygens’s approval of his new invention and stresses the fact that his own device does not rely on a geometric principle but on a completely mechanical one.^24^ For this reason, he adds, he finds it strange that no artisans have noticed this possibility before. The importance of the new invention’s purely mechanical approach is also emphasized by Leibniz in a note found in *Leibniz-Handschriften zur Technica*, published posthumously in 1718 as a book review in the *Journal des Trévoux*.^25^ We will return to these notes below.

Leibniz was not only concerned with new timekeeping principles, writing down descriptions, calculating, drawing, and planning future clocks, he was apparently also busy making pendulums and pendulum bobs with his own hands. He discovered a new way of using cardboard to create clock parts, although it is not known whether he used these parts only as prototypes for experiments or for manufacturing real working clocks.^26^ Leibniz’s long-term relationships with a number of different clockmakers whom he commissioned to work on his grand calculating-machine project are also essential to note, among them Ollivier (first name unknown), Georg Heinrich Kölbing, and Adam Sherp.^27^ During their long-term relationship, Leibniz worked closely with Ollivier in workshops, received detailed reports on his progress while away, sent him models and parts to work on, argued with him on the feasibility of some of his ideas, and quarreled with him over payments and deadlines.^28^

To better examine these convoluted relationships between Leibniz and clockmaking, in Section 2.1 we examine Leibniz’s views on clockmaking in general, as well as some of his general references to clockmakers around him, including his opinions of their work. In Section 2.2, we move on to consider Leibniz’s early interest in clockmaking, in particular his reading of Huygens’s *Horologium oscillatorium *and his recurring report on finding a new principle of timekeeping. We examine the short piece Leibniz published in *Journal des sçavans* (1675) and attempt to determine the extent to which Leibniz was acquainted with the mechanics of clocks and artisanal practices of clockmaking. We then show how Leibniz’s claim that artisans should have “stumbled upon” his mechanical principle a long time ago acted as a form of implicit praise for his own new discovery.

### 2.1 Leibniz on Clockmaking and Clockmakers

Rather than just another craft, clockmaking had a somewhat elevated status for Leibniz. This can be ascertained in a letter he wrote to Huygens in October 1690 where he declares how important Huygens’s book on the pendulum clock *Horologium oscillatorium* (1673) was for him: “your excellent work on pendulums was one of the greatest reasons for the progress that I have perhaps made since then in these kinds of sciences.”^29^ Clockmaking and clocks were conceived by Leibniz as essential for the advancement of the sciences, most notably astronomy and naval navigation.^30^ This emphasizes how the importance of a craft for Leibniz was derived less from the craft itself and its manufactured objects than from its contribution to the higher purposes of more theoretical domains—a claim discussed below. Moreover, Leibniz praised Huygens’s pendulum clock as the highest achievement in the history of the art of timekeeping, claiming that Huygens should in fact be credited as the true inventor of the clock precisely because he was the first to have based horology upon firm mathematical principles.^31^

Leibniz was very much aware that artisan clockmakers helped to execute such inventions, noting, for example, that it was the clockmaker M. Thuret who built the “first spring watch” designed by Huygens.^32^ One may interpret Leibniz’s statements as pointing to a dichotomy between natural philosophers and artisans, since the artisan is mentioned only alongside the name of a “proper” inventor with whom he was working.^33^ The makers who do receive the full honor of being presented on their own are the ones who contributed to horology by means of arithmetic or geometry and were assisted by additional artisans to construct their inventions.^34^ Moreover, Leibniz generally had a low opinion of German clockmakers, whom he felt were not sufficiently open to new ideas.^35^ In the book review published posthumously in the *Journal des Trévoux*, he remarks on his inability to manufacture his invention of the double-spring watch in Germany:

“I have sometimes thought of realizing this invention, which promises new and fairly considerable advantages; but I always lacked the assistance of a good master who had the will to work on it; ordinary workers, especially in Germany, have no desire to deviate from their routine.”^36^

This critique was not directed only toward German artisans, however; one hears a similar tone when Leibniz describes his work with Ollivier, a Frenchman who was no ordinary clockmaker but one that Leibniz had chosen out of many to help him work on his groundbreaking calculating machine. Nevertheless, as Leibniz writes, even he “protests that he prefers to make his living easily in the ordinary way, rather than to embark for nothing in an enterprise full of disquiet and risk.”^37^

Having said that, we should be careful not to take Leibniz’s views on his inability to find help for the double-spring-watch project at face value. Although he mentioned his invention of the double-spring watch numerous times, it seems that the reason for mentioning it so often was due to the principle underlying it, viewed as a new principle of equality of durations of time. The motivation behind the repeated mention of his invention may well have been the need to cement his priority alongside Huygens’s by then much celebrated principle of isochronism, termed “geometric” or “physical” by Leibniz. There is no evidence that Leibniz ever tried to build the watch, and his claim that he was unable to find help might have been a way to deflect attention away from this fact. More importantly, it seems to have been the purely mechanical nature of his new principle that he thought was so appealing, making it in fact comparable to Huygens’s achievement. If that were the case, then turning to blame the artisans’ unwillingness to take risks for the incompleteness of this project does not make any sense and may even be considered contradictory. We turn now to examine this issue more closely.

### 2.2 The Spring-Governed Watch and a New Principle of Time Keeping

Leibniz repeatedly declared that his unique contribution to the science of horology was his discovery of a new principle of timekeeping, or as he termed it, a new “principle of equality.”^38^ Leibniz is referring to Huygens’s geometric solution of a so-called isochronous or tautochronic problem, or the problem of the sameness of time, celebrated in his 1673 book *Horologium ocillatorium*.^39^ Huygens’s construction of a pendulum swinging between cycloid-shaped bars was in fact the first constructed machine whose goal was to divide time into equal units of duration, based on a mathematical argument. This argument came down to the discovery that, under the assumption of gravity, a mass would roll down along the cycloid curve isochronously; that is, it would always take the same time to fall to the bottom no matter from which height it was released. That meant that a pendulum oscillating along a cycloid, slowly losing its height (or amplitude) due to the friction of the air, would nonetheless take exactly the same time to finish a full cycle of oscillation, irrespective of the width of the swings. Apart from the obvious technological breakthrough such an instrument exhibited, it also broke conceptual ground, and may be seen as the first mathematical-mechanical construct of a self-standing, quantified measure of time.^40^ Nevertheless, although Leibniz mentions that at least in the last quarter of the seventeenth century most clockmakers were imitating the cycloidal pendulum, its use and production soon declined and was replaced by devices relying solely on a regular pendulum with escapement mechanisms, which were easier and cheaper to produce, and contributed to accuracy more than the cycloidal shape of the pendulum itself.^41^

Since Leibniz insisted on the mechanical nature of his own principle of equality, which was based on a spring mechanism (see below and Section 2.2.1), Huygens’s pendulum brings out another important facet of the mechanical-geometric distinction. In early-seventeenth-century mathematics, the cycloid was considered a mechanical curve, which could not be expressed algebraically, and was therefore excluded from Cartesian geometry.^42^ We will later show that for Leibniz the cycloid, along with other curves as well as various artisanal practices, served as examples of a hidden geometry (*Geometria arcana*), which geometry should aspire to encompass. Indeed, as illustrated by Probst (2012), Leibniz’s later definition of geometry also included the transcendental domain, formerly excluded by Descartes as mechanical. Probst points out that in 1674 Leibniz classified curves into two types: geometric and mechanical. Geometric curves were those that could be drawn in one stroke, while mechanical curves were drawn only pointwise. Hence, artisanal machines moving along geometric curves should also be included in this hidden geometry.

Although there is no evidence that Leibniz wrote on his new principle of timekeeping before 1675, he later claimed that in 1671 he had already thought of a spring-based mechanism that could be used to divide and measure time into equal units. The equality of the vibrations of springs was verified for him not so much experimentally but rather by an analogy to the simple fact that “equally tensed” *strings* will “always give the same tone”:^43^

“But there would still be something to be said about the nature of the vibrations of the springs, the equality of which is verified by those of the touched strings, which always give the same tone when they are equally tensed.”^44^

Leibniz seems to be wondering in his 1718 book review whether a geometrical presentation as elaborate and difficult as Huygens’s is really needed for achieving isochronism, since a simple and direct meditation on a vibrating string can lead to the same conclusion. Leibniz claims that pure temporal repetition, which came to be known as harmonic oscillation, is in fact self-evident and found everywhere in nature, especially in elastic materials. The same point—that of contrasting his principle to Huygens’s “physical” principle—is found again later in the paper:

“When Mr. Huygens published his spiral vibrating spring,^45^ I published a little after in the *Journal des sçavans* another principle of equality, which is not physical, such as the supposition of the equality of the vibrations of pendulums, or of springs, but purely mechanical, consisting of a perfect restitution of what must vibrate, since then the vibrations are equal, for they are precisely the same.”^46^

The term “physical” in this context signifies for Leibniz that Huygens’s principle is based on the observation of a natural phenomenon, in this case the swings of the cycloidal pendulum or the vibrations of a spiral spring, whereas his principle is mechanically constructed and not found and observed in nature itself. In a letter he sent to Oldenburg in May 1675, for example, Leibniz explains that Huygens’s principle is derived from “oscillations made by nature herself” (*oscillationes ab ipsa natura*) (Oldenburg 1977: 305).^47^ A similar claim is also made at the beginning of the article for the *Journal de sçavans* in March 1675: Huygens’s principle “depends on physical observation, whereas mine is based on a purely mechanical reflection, which is easy enough.”^48^

This passage takes us back to the aforementioned draft of the letter Leibniz added to the copy of the *Journal des sçavans *article he sent to Oldenburg. There, the principle behind the new invention is described as something that should have been discovered already by the artisans—although this passage was deleted from the letter eventually sent on March 30, 1675.^49^ In the draft, Leibniz writes:

“Huygens himself, who recently, as you know, published that elegant application of oscillating springs to watches, very much approved of mine as being novel and purely mechanical and dependent on no physical experiment or geometric demonstration, so that it is surprising that the artisans did not stumble upon it long ago.”^50^

Artisans seem to play a peculiar role in Leibniz’s self-praise here. He is surprised no artisans have ever “stumbled upon” (*in eam incidisse*) this principle, or at least utilized it in their creations. But this surprise is also rhetorical: the claim that artisans should have already come up with this principle is meant to highlight the unique character of his discovery. It is not quite so that the work of artisans here represents a peak of knowledge for Leibniz. The point is rather that what is described as a very fortunate stumbling upon a principle is praised as highly as a theoretical philosophical argument leading to the same achievement. Both elicit a new technological conceptualization, and the artisanal practice even supplies the scale for praising the achievement in general. Moreover, when Leibniz writes about the mechanical reflection he applied in order to arrive to his principle, he underlines that this has “not been taken notice of, for want of the art of combination, the use of which is far more general than that of Algebra.”^51^ This view may imply that algebra might not suffice here to conceptualize Leibniz’s ideas, a view that will be seen as well later. It also might open a way to rethink Leibniz’s general views on theoretical, mechanical, and artisanal knowledge, as we will do in Section 4.

Leibniz believed that most scholars and inventors simply followed Huygens’s discovery, eventually imitating it. He himself had a different source of inspiration, a new “principle of discovery,” equated earlier in the unsent draft with the ability and knowledge of artisans. The draft of his letter concludes with a plea that Oldenburg publish his “invention” in his journal *Philosophical Transactions*, as indeed occurred a month later.^52^ In the deleted paragraph, Leibniz depicts his new principle of discovery as superior to the meditations of other clockmakers of his time:

“It usually happens that, after one excellent invention has been published, such as the oscillatory clock, the meditations of others, as if captivated by and fixed on this one, have something of imitation that is not easily removed: the mind, as if preoccupied with this, is rarely raised to a different principle of discovery. As to the discovery itself, you will judge from the description and figure, and if it seems to be worthy, you will insert it into your *Transactions*.”^53^

Leibniz’s attempt at convincing Oldenburg to circulate the new invention in his journal relies mainly on the unique, artisan-like nature of his discovery, since, unlike other clockmakers and inventors, he had not fallen into the habit of simple imitation. Rather, his discovery had a completely different source: neither geometry nor great geometers such as Huygens, but a source of inspiration available to artisans. On the one hand, the figure of the artisan can be seen here as representing a form of simplemindedness, a sort of knowledge that does not require much sophistication or prior theoretical preparation.^54^ On the other hand, Leibniz viewed his new principle as better than Huygens’s precisely because it was mechanical rather than geometrical or physical.

Judging by the number of times Leibniz referred to his discovery of his new principle, his mention that he even had witnesses that it “was known” (*nota fuit*) to him before Huygens published his own spring watch,^55^ and his claim that he never actually built (or attempted to build) a device utilizing this new principle, we may assume that at least in this case the theoretical achievement was probably more important to him than its construction. Nevertheless, Leibniz did publish the details of the plan for construction, which we will now briefly examine.

#### 2.2.1 The Description of the Double-Spring Watch

**Fig. 1:**
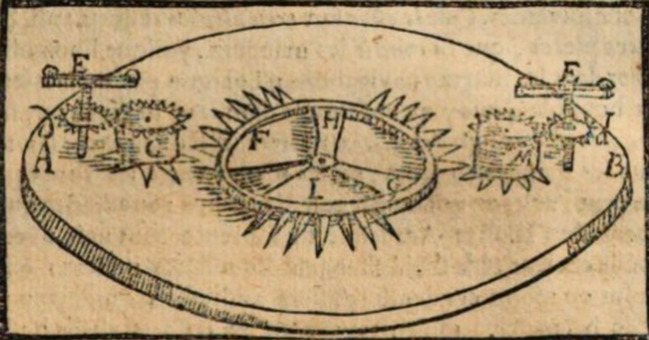
Illustration of the double-spring mechanism published in *Journal des sçavans*, March 30, 1675

The March 1675 *Journal de sçavans* opens with Leibniz’s declaration celebrating his new principle of equality. He then states how he “inferred” that if one would employ two springs, it would not matter if the first overrides the other “more or less quickly,” provided that it opens again “before the other finishes stretching again” and that “this game will always continue uniformly.” Finally, he concludes that in “letting pass at each return or period of these two springs, a tooth of a certain wheel driven by the ordinary movement, and which counts the seconds or other parts of time equal to the periods, we have a clock or watch such as we may desire.”^56^ From here, Leibniz goes straight to a technical description of the proposed mechanism, which is presented as follows: *AB* marks a plate of a clock, and *C* and *M* are toothed barrels in which small spiral springs are locked. The teeth of the barrels “take the teeth” of the “pignons” *D* and *D*, which carry the balancing wheels *E* and *E*. The other teeth of the barrels (*C* and *M*) take the teeth of the central pivot wheel, *FG*. Only after presenting most of these details did the editor of the *Journal des sçavans*, Jean-Paul de La Roque, add the following drawing of the two-spring mechanism, the only illustration that appears in the paper (see Fig. 1).

Leibniz goes into further detail on how each part of the mechanism interacts with neighboring parts. We will not present all these details, but simply note that Leibniz’s treatment of such issues shows he had a genuine understanding of how timekeeping mechanisms worked and was not content with merely presenting theoretical principles.^57^

* * *

Before moving on to present Leibniz’s views on another kind of artisanal knowledge—that of textile artisans—and on how the latter’s machines may have embodied secret mechanical-mathematical principles, we would like to summarize the different relations between artisanal, mechanical, and geometric knowledges presented above. Earlier on, we presented the straightforward opposition between artisanal and theoretical types of knowledge, which in Leibniz’s work is presented in the form of a clear hierarchy: Leibniz as both philosopher and inventor takes risks, while the artisan sticks to the familiar. Artisans are characterized as helpers in charge of bringing an already finalized idea into material existence, and ultimately, for Leibniz, the goal of timekeeping instruments is to advance theoretical knowledge, most notably astronomy and naval navigation. However, we have also considered further distinctions that might blur this sharp opposition. Indeed, Leibniz introduces a distinction between geometric and mechanical principles of timekeeping; he claims that his newly discovered “principle of equality” is as good as, if not better than, Huygens’s geometric principle, since his principle is “purely mechanical” and should have been thought of or discovered by artisans.

But Leibniz opposes mechanical knowledge and geometric knowledge in yet another, somewhat different way: he addresses the Cartesian distinction between mechanical and geometric curves in many different texts.^58^ In a strictly mathematical sense, this notion is linked to Leibniz’s work on the transcendental, which would amount to non-algebraic, mechanical (in the Cartesian sense) curves such as the cycloid—upon which Huygens’s geometric principle is based—and to transcendental functions such as *sine*. The examples that Leibniz supplies for this new geometry come from both mechanical curves and different artisanal practices, mechanical inventions, and natural phenomena. Here, it is already clear that the class of mechanical curves, which can only be drawn pointwise, as Leibniz noted in 1674, is not equivalent to the curves along which the artisanal machines move, which are considered geometric (see Probst 2012). We will treat this more closely in the following sections, but for now we will raise a question regarding Leibniz’s use of the term mechanical. We see a clear change of meaning of this term between Descartes and Leibniz: could one link Leibniz’s understanding of the mechanical with the task of expanding geometry? We will now turn to this and other questions regarding the relationship between mechanical, geometric, and artisanal knowledges as they are exhibited in Leibniz’s writing on and interaction with textile crafts.

## 3. *Geometria Sartorum*: Leibniz on Weavers and Textile Artisans

Leibniz’s claim that it is remarkable that artisans overlooked the mechanical principle “of equality in the clock” stands in contrast to Huygens’s geometric principle, which artisans are not even expected to understand. Did Leibniz consider other handworkers and artisans—for example, weavers, spinners, knitters, and so on—in this way? The answer is yes but is nuanced, since textile artisans also had a unique place in Leibniz’s thought. And while we will not examine all of the passages in which Leibniz referred to such artisans, we will review in this section several episodes that exemplify his unique relationship to textile workers.

As mentioned in the introduction, this unique relationship can already be seen in May 1673, when Leibniz wrote to Christian Habbeus, detailing his impressions of the artisans of Paris and describing not only the stocking frame, a machine that mechanized knitting, but also clockmaking.^59^ In April 1675, Leibniz wrote a short note entitled “Geometria Amoenior/Subjicienda Geometriae arcanae” (A More Pleasing Geometry/Subject to Hidden Geometry), containing a series of references to and reflections on topics already possibly researched but not necessarily included within the domain of geometry. Among these topics is the “Geometria Sartorum” (geometry of tailors), or the “the craft of boys, through which they extract [raise up, draw out] the strings which are entangled with their fingers.” Another such topic is “the art of textiles.”^60^ In the same note, Leibniz also mentions Huygens’s pendulum.

As with clockmakers, Leibniz corresponded during the 1670s and ’80s with textile projectors and textile machine inventors such as Johann Daniel Crafft (1624–1697), who wrote to Leibniz on the stocking frame and the ribbon loom, a machine that partially mechanizes the simultaneous weaving of several ribbons.^61^ Concerning the latter, Leibniz noted in 1680 that Crafft “now has a miracle in his hands, [and] it seems that a new lesson will be given to the whole world, practicing reason [*ratione usus*], for, of its kind, it [the ribbon loom] is the proper philosophers’ stone.”^62^ That Leibniz ascribes to Crafft’s loom the same capabilities as the alchemical “philosopher’s stone” may show his estimation for such a machine, which enables the “blending” or combination of simple substances (in this case, threads) into an intricate fabric. Crafft announces in November 1681 that his ribbon looms are “per se in perfection” and can be used for “silk weaving.”^63^ These knitting and weaving machines are also noted by Leibniz in September 1688, as discussed in the introduction.^64^

These references, though presented in a very condensed form, already show that Leibniz had a genuine interest in the work and practices of textile artisans. Elaborating on this interest in Section 3.1, we will examine Leibniz’s discussions on the stocking frame and the ribbon loom—which emphasize how geometric and mechanical knowledge and principles were considered with respect to textile-related artisanal knowledge—and link these discussions with the aforementioned reflections on horology. We then move on in Section 3.2 to review Leibniz’s views on hidden geometry, a project whose aim was to both extend geometry beyond its Cartesian limits and expand geometry to include artisanal practices.

### 3.1 Geometric or Mechanical Principles? Leibniz on Textile Machines

How did Leibniz consider textile machines? As we will see, his considerations oscillated between ascribing to such machines the general characterization “geometric,” without specifying how they are actually geometric, and more explicitly stating the mechanical laws and mathematical theorems according to which they function.

#### 3.1.1 The Stocking Frame

In the preface to his 1676 *De quadratura arithmetica*, Leibniz describes the stocking frame—discussed extensively elsewhere (Friedman 2023a)—as a “weaving machine” (*Machina textrice*) invented by a “geometric genius.” He then equates it to his own “arithmetical instrument, which carries over all the work of the mind to wheels.”^65^ As we do not wish to repeat the analysis presented in previous works, it is enough to emphasize that such a conception posited artisanal *manual* knowledge—here, of knitting—as inferior to the geometric knowledge embodied in the machine. What this geometric knowledge is will be discussed later, but, as already noted in Section 2, this characterization of knowledge as geometric points to a certain type of knowledge that is not accessible to artisans—it may, however, be associated with the inventors or constructors of machines. At the beginning of his preface, Leibniz states that “one hears arguments about the worthlessness of geometry … [concerning] its practice. … very many useful things have been invented formerly, … [but] these things are expected from [the study of] geometry.”^66^ Such “things” and inventions are described later in the text, among them the stocking frame and Huygens’s pendulum clock.

This dichotomy between artisans and geometers is in fact complicated by Leibniz himself, since geometric knowledge is contrasted here with “combinatorial” knowledge. Leibniz makes a comparison between the geometric and combinatorial genius, noting that the latter’s “inventions are simple and are entirely explained in a few words, since they are, for the most part, in need of observation rather than reflection…”^67^ Can one suggest that artisans may have also belonged to this group? If in the early modern period artisanal knowledge was mainly transmitted orally, this is reflected in Leibniz’s statement about combinatorial knowledge being “explained in a few words,” in the sense that it did not need to be written down. This stands in contrast to how the machines that mechanized these artisanal practices are considered. If the stocking frame was invented by a “geometric genius,” those who practice geometry are thus characterized by a “mathematical genius [talent; *ingenio*] [acquired] through prolonged and difficult [efforts]—not by a skill of trying or by the luck of guessing, but by a certain vigor of the mind.”^68^ Thus, one again sees a contrast between the geometric and combinatorial genius.^69^

After describing the stocking frame in *De quadratura arithmetica* as a machine invented by a geometric genius, Leibniz goes on to explain how Huygens should be seen as both a geometric and combinatorial genius. In contrast to the stocking frame, and perhaps because Leibniz knew Huygens personally, Leibniz explains why Huygens should be considered a genius—namely, because he was able to finish Galileo’s work on the isochronism of the pendulum by completing its two unsolved tasks: “the application of the pendulum to the clock, by which the labor of counting would be removed, and the invention of a curved line, … for the arc of a circle could not perform this, hence the repeated long vibrations of the pendulums were undoubtedly erroneous.”^70^ Leibniz is referring to the known fact, already perceived by Galileo himself though never demonstrated mathematically or explained, that the oscillation of the circular pendulum is not truly isochronous and becomes less accurate in longer oscillations.^71^ Huygens’s achievement, as noted above, was the mathematical solution for finding the truly isochronous curve describing the swing of the pendulum (or the tautochrone) and showing it was the cycloid, as well as constructing actual clocks based on this discovery. For the former achievement, he is considered a geometric genius; for the latter (that is, the constructions), a combinatorial genius: “one [achievement] was to be performed by the combinatory art [genius], the other by the geometric genius, both of which Huygens completed beautifully in his immortal work.”^72^ Here, Leibniz sums up the discussion by saying that all his examples amount to sufficient evidence for the existence of a “deeper [more profound] geometry”;^73^ all pertain to the need to expand geometry into a new but still undefined inventory of transcendental curves and non-algebraic functions.

#### 3.1.2 The Ribbon Loom

**Fig. 2:**
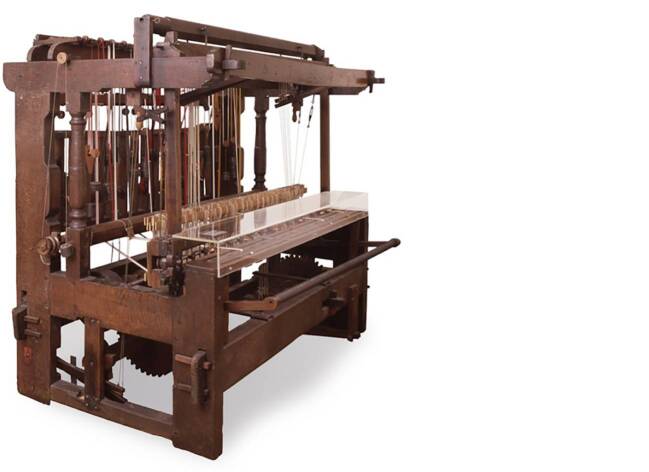
A wooden ribbon loom from Basel, 1776 (H 200 cm × W 240 cm × D 140 cm). Inv. 1881.166. © Historisches Museum Basel, Maurice Babey

Moving on to the second textile machine Leibniz discussed, he considered ribbon looms (see Fig. 2) first and foremost as increasing the production of goods. In his 1687 manuscript *Discussion d’une question utile et curieuse*, Leibniz asks whether “it is necessary to admit the introduction of instruments, which reduce work, and by means of which one man can do as much as several men.”^74^ He then lists several cases in which weaving machines—referring implicitly to ribbon looms (and explicitly to *Mühlstühle* and *Schnürmühlen*)—were banned from use for fear that they would lead to unemployment, though for Leibniz the advantage of these machines and instruments was the resulting increase in human capacities (“l’augmentation de son pouvoir”).^75^ However, bans issued in various cities in the Holy Roman Empire during the seventeenth century were based on a fear that artisans would not only lose their jobs but also starve to death.^76^ Leibniz dismissed such anxieties.^77^ Nevertheless, such bans and edicts were scarcely implemented at the beginning of the eighteenth century; at the end of the seventeenth century, the economic decline of the manual braid and ribbon weaving continued due to cheap ribbons sold in German markets and as technical progress of the ribbon loom could not be stopped in the long run. Nevertheless, as Reinhold Reith stresses (2000: 36–40), the narrative of hostility to technology per se should be revised because the productivity of the ribbon loom was not as high as one might think, and until the nineteenth century, the ribbon loom produced mainly simple belts.

As already noted, Leibniz lists his calculating machines, ribbon looms, and clocks as great “inventions” alongside the stocking frame. Moreover, while geometric knowledge was associated with the stocking frame, the principles according to which ribbon looms operated could be considered as oscillating between mechanical and geometric. When around 1706 Leibniz discusses an “instrument suited to more ingenious weavers” (*textoribus ingeniosoribus instrumentum*) that weaves floral figures and is later revealed to be the ribbon loom, his principal concern is how the friction of this machine can be reduced.^78^ He notes immediately afterward the discovery made by Ole Rømer in 1674 that cogwheels with epicycloidal teeth revolve with a minimum of friction. Indeed, as Joel Mokyr emphasizes, this discovery was not only relevant for ribbon looms: “Clockmakers wanted to know the optimal shape of the teeth on gear wheels that would minimize friction: the mathematicians Ole Roemer and Christian Huygens were able to show that the epicycloid was the curve satisfying this condition” (Mokyr 1992: 75).^79^ But when describing Rømer’s discovery with its many mechanical implications, Leibniz notes that it was made “non sine profundioris Geometriae auxilio” (not without the aid of deeper [more profound] Geometry) (Gerland 1906: 118). Here, Leibniz emphasizes not only the geometric knowledge which is supposed to be at the basis of the mechanical ribbon loom but this geometry’s more profound character, which again points to its hidden dimension.

### 3.2 Leibniz’s Secret Geometry: Mechanical Curves, Textile Machines, and Pendulums

Leibniz’s call for the use or discovery of deeper or hidden geometric principles is also found in other writings from the 1670s. He reflects upon how one may expand geometry in the aforementioned note from April 1675 entitled “Geometria Amoenior/Subjicienda Geometriae arcanae.”^80^ This note may either set out a plan for future topics to be investigated by geometry (or by Leibniz) or list domains to be included within geometry. The opening sentence already sets out geometry’s task: to produce explanations concerning “figures” that appear in both nature and technical artisanal practices (*ars*) in a unique way.^81^ But here one should also note what Leibniz erased. The opening sentence, including the erasure, reads: “Geometriae est explicare figuras quas natura et ars nobis non cogitantibus producit.”^82^ What does Leibniz mean by “nobis non cogitantibus” ([even] without us realizing [thinking about] it)? Does he implicitly refer also to artisans, who are not in a position to understand geometric reasoning?

Addressing, among other things, geometric and mathematical objects, Leibniz observes the following in this note: “The task of geometry is to explain figures that nature and the arts produce through unique reason: … Geometry of tailors … on the craft of boys, by which they draw out threads entangled around their fingers … on the art of textiles. On the entire kind [genus] of fabrics [looms, warps], velours, and so on. On the silk-stocking instrument.”^83^ Textile-related topics are not the focus of this list, since the list itself has no real focus; indeed, it refers to a broad range of subjects and domains. Leibniz also mentions works written by various mathematicians and natural philosophers, among them Huygens, whom he refers to three times, the third referring explicitly to the pendulum: “De Mensura constante per pendulum, Moutoni, Hugenii, Buratini.”^84^ All three of these thinkers suggested grounding the measurement of the earth and the constitution of a universal length unit on the length of the second pendulum, though in different ways.^85^

Nevertheless, in this list there are no references to particular (that is, named) artisans—though Leibniz does mention specific machines, including the stocking frame. Moreover, it seems that the geometry referred to (either of textile machines or of measurement by the pendulum) is secret (*arcana*); that is, it is something one would expect natural philosophers to reveal or formulate rather than artisans. Such a view, at least concerning textile practices, may be also noted when years later Leibniz copied Jungius’s manuscript, *Texturæ Contemplatio* (Contemplation on Weaving), where Jungius attempted to formulate definitions and theorems in a geometric manner concerning various weaves.^86^ However, another note written by Leibniz several months before “Geometria Amoenior,” shows that hidden geometry’s meaning may have been broader.

As early as December 1674, Leibniz mentioned a secret geometry in his note “De progressionibus et geometria arcana et methodo tangentium inversa.”^87^ As Michel Serfati has remarked, here Leibniz examines

“curves given by a condition on their tangents … lead[ing] to a differential equation. The latter thus requires … a calculation of primitive curves of the equation, for which Leibniz first proposes a method where the quadrature leads to an algebraic result, and then judiciously expresses a reservation: ‘If no analytical curve happens to succeed, the question would be to look if it is not possible to find a transcendent geometrical curve, as one of the kind of trochoids, evolutes, or helices.’^88^ This time, it is thus a question of a ‘transcendent curve,’ although it remains in a vague sense” (Serfati 2018: 4).

While the concept of a transcendental curve does not appear in “Geometria Amoenior,” Leibniz was already thinking about this term during this period, as Serfati underlines.^89^ Leibniz obviously means that one has to consider the mechanical curves that Descartes excluded from the domain of geometry and introduce them into it.^90^ One of the ways to do this is to examine the various machines of artisans.

This is not the last time Leibniz describes this new geometry, which should include in its domain curves that arise during mechanical investigations (for example, the tautochrone [cycloid], the epicycloid, the tractrix, and so on), as secret or hidden. Two additional examples may be given.^91^ First, it should be mentioned that around late 1678 or 1679 at the latest, Leibniz copied Jungius’s lecture notes from 1644 on phoranomics (the study of local motion or of moving geometrical objects).^92^ In these lecture notes, Jungius presents various curves—among others, the conchoid, the cycloid, or the epicycloid—as both phoranomical and geometrical.^93^ Second, in his well-known 1686 text *De Geometria recondita et Analysi Indivisibilium atque Infinitorum* (On the Hidden Geometry and the Analysis of Indivisible and Infinite Quantities), in which Leibniz publishes the proof of the fundamental theorem of calculus, he talks about such mechanical curves as needing to be included in the realm of geometry.^94^ What appears in all these texts (from December 1674, April 1675, and 1686, as well in the copy of Jungius’s notes on phoranomics) is the call to expand geometry. In this sense, the geometry of tailors, the geometric research on the pendulum, and the consideration of mechanical curves all reveal this hidden geometry. One can claim that the mechanical means mentioned here—either mechanical curves or artisanal practices—should not remain hidden but be considered geometrically.

In contrast to our discussion on clockmakers, Leibniz does not even expect that (German) textile artisans—at least those who are *not* involved either in the construction or in the use of such machines—would be in a position to stumble upon the mechanical principles underlying the textile machines. As noted above, Leibniz saw Jungius (and not the artisan) as suitable to elevate the research on textiles to a contemplative activity formulated with theorems and definitions.^95^ Moreover, only those who construct such textile machines, or more specifically the stocking frame, are presented as geometric geniuses.^96^ As Leibniz himself notes, there are also geometric geniuses “who do not know that they themselves are geometers, although they reason seriously and profoundly by means of that style of argumentation that they understand.”^97^ This is an explicit reference to artisan-constructors, who reason in a geometric way, yet are not even aware that they do so. In that sense, while Leibniz does not claim that artisans stumble upon geometric or mechanical principles, he does argue that they act according to them, though do not understand them. This is the essential point here that Leibniz states explicitly: there is a distinction between the various types of reasoning.

However, the distinction between geometric reasoning and mechanical reasoning is complicated by Leibniz and becomes somewhat vague. On the one hand, the new—that is, transcendental—curves (epicycloid, cycloid, sine, and so on) are hidden or secret and should be included in geometry. Leibniz also advocated Jungius’s view—that weaving practices (or products) may be formulated in a geometric manner. On the other hand, such curves manifest themselves in the mechanical (textile) machines themselves—in our case, the ribbon loom. This conception should be considered together with Leibniz’s 1676 view of secret motion (*arcanis motus*). As Mogens Lærke has shown, in *De arcanis motus* Leibniz wanted to reduce the secret of mechanics—and how such machines move—to “pure geometry” (Lærke 2015: 125). In early 1676, Leibniz wrote to Claude Perrault that he “now thinks [he] can account in a satisfactory way for the laws of movement by means of entirely geometric demonstrations, without making use of any supposition or principles of experience.”^98^ However, as Lærke notes, in late 1676 Leibniz began to depart from these conceptions (Lærke 2020: 36–55). In contrast to the discussion on the pendulum, Leibniz does not explicitly differentiate between the geometric principles according to which the ribbon loom (or the stocking frame) works, and the mechanical principles which guide the construction of the machines (reducing friction).

## 4. On the Extraction of Secrets and the Expansion of Geometry

Notwithstanding his oscillating views concerning artisans’ knowledge, examined in the previous two sections, Leibniz held that (Parisian) artisans did possess an inventive and secret knowledge embodied in the machines they built. This view is presented explicitly in Leibniz’s May 1673 letter to Christian Habbeus, mentioned above. In it, he describes his impressions of the remarkable artisans of Paris,^99^ mentioning, for example, the stocking frame.^100^ More generally, as noted in the introduction, he describes his encounters with local artisans in Paris along with his and the King’s wish to “extract their secrets and inventions.”^101^

Knitting with the stocking frame is not the only artisanal practice that is mentioned. Leibniz also cites artisans who make crockery or pottery (*une certaine sorte de vaisselle de terre*), or those who work “well with iron … who have the secret of throwing iron into shapes, and then filing it, which is not common practice.”^102^ He finishes this list by noting that “there are an infinite number of curiosities, such as in goldsmithing, enameling, glassmaking, clockmaking [*horlogerie*], leather manufacture, pewter pottery, which abroad [by our foreigners] is not for the most part taken notice of …”^103^ The mention of *horlogerie* is essential, since here as well Leibniz underlines that German artisans (“nos estrangers”) do not pay attention to such artisanal works and machines. This view echoes the partially erased sentence from the April 1675 note in “Geometria Amoenior,” when Leibniz mentions geometry’s task of explaining various matters to those “who do not realize” geometric reasoning. In this sense, artisans—or at least French ones—are presented as possessing secret knowledge, though it is not clear whether they are aware of such aspects of this knowledge, or if any (geometric or mechanical) principles can be extracted from their “secrets and inventions.” As elaborated below, we argue that the secret knowledge attributed to artisans reflects Leibniz’s notion of a hidden geometry, which he posits as a domain for the expansion of geometry.

Leibniz’s wish to extract artisans’ secrets and inventions reflects a tension between how artisanal knowledge is embodied and practiced and how it may emerge or be formulated as geometric or mechanical, a tension also presented by Leibniz in various writings. These reflections illustrate Leibniz’s ambivalent relationship with artisanal knowledge: rather than encouraging artisans to record their own knowledge (or attempt to write down their own mechanical or even geometric reflections), he considers that the codification of technical practices upon which technological progress depends is a job more suited to (natural) philosophers and mathematicians (Young 2017: 542).^104^

What, then, was the social background against which Leibniz developed his reflections on artisans and their knowledge? Was his differentiation between types of knowledge and between types of artisans unique, or was this common during the end of the seventeenth century? In the following section, we discuss the distinction between mechanical and geometric knowledge in a wider seventeenth-century framework, examining how artisans were viewed in late-seventeenth and eighteenth-century France, and how Leibniz in particular saw them as figures possessing secret knowledge.

### 4.1 On Artisans, *Artistes*, and Geniuses

Although Leibniz did not employ this terminology, his differentiation between the various types of knowledge is related to the rise of a unique type of artisan: the *artiste*. According to Paola Bertucci, the term first emerged in the context of practical alchemy and chemistry at the beginning of the second half of the seventeenth century, challenging earlier prejudices about artisans. It was employed to emphasize that intelligence and *esprit* were required to perform their professions. Indeed, around 1700, a broader definition viewed the *artiste* as an artisan who, “in addition to manual skills, could also boast of that ineffable essence of French intellectualism, *esprit*. … As an artisan with *esprit*, the *artiste* consistently presented himself as superior to other craftsmen because his work did not consist of rote practice but resulted from his ingenuity, an ability to combine practical skill, creative design, and inventive intelligence.” Bertucci further notes that how Denis Diderot emphasized that while artisans “were ‘workers who practice the mechanical arts that need the least intelligence,’ … *artiste* was a ‘noun that one gives to workmen who excel in the mechanical arts that need intelligence’” (Bertucci 2017: 4). This explains why in Diderot’s *Encyclopédie* there are numerous descriptions of artisans and artisanal work. Bertucci also explores the Societé des arts, founded in 1728, whose members differentiated themselves from mere artisans by proclaiming themselves *artistes*, thus showing an increased interest in the mechanical arts presented at the Académie royale des sciences.

Here, it is important to emphasize that Bertucci does not blindly follow Pamela Smith’s conception of “artisanal epistemology” (Smith 2004). Bertucci underlines how the mechanical arts and machines gained importance in France during the first half of the eighteenth century (Bertucci 2017: 176–205). She also notes that the knowledge of *artistes* was not typical of all artisans but epistemologically differed from that of other practitioners. In this sense, Bertucci explicates what we have already seen in Leibniz: numerous scholars and natural philosophers of the seventeenth century were fascinated by artisanal practices, even while distancing themselves from them. Smith describes this attitude as a “combination of lively interest and continuing low regard for handworkers” (Smith 2022: 168). This might stem from the fact that during the sixteenth and seventeenth centuries, most artisanal practices were transmitted orally: artisans “did not need written texts in order to learn or transmit knowledge, for craft knowledge was efficiently transmitted by means of apprenticeship and disseminated relatively rapidly by the embodied knowledge moving in the artisans themselves” (Smith 2022: 95). This stands in contrast to the *artiste*, whose knowledge was openly shared for the public good.

Bertucci’s research on the category of the *artiste*, located between the artisan and the savant, echoes to a certain degree how Leibniz viewed the various artisans who excelled in the construction of machines, be they textile machines or clocks and pendulums. However, it must be stressed here that the term *artiste* is mainly used for French artisans, whereas Leibniz held a low opinion of German artisans. Moreover, he questioned the anxieties of (German) artisans who failed to recognize that the use of machines in general would lead to an increase in productivity and reduction of costs, manpower, and worktime.^105^ While the social background of these two groups is indeed different, common to both is Leibniz’s conception of the constructors of such artisanal machines. Hence, as noted in the introduction, Leibniz’s claim that the artisan’s knowledge is secretive is perhaps due to the fact that it was not known to the public; nevertheless, the artisan did have unique access either to materials found in nature or to machines. Another way to view this claim, however, is by pointing to a mechanically or mathematically based knowledge, which perhaps unbeknownst to the artisan is possibly known to the *artiste*. In any case, this already shows how Leibniz and his interaction with artisanal knowledge do not neatly fit into any of the known categories that attempt to grasp the place of artisanal knowledge in seventeenth-century natural philosophy.

As mentioned above, there have been numerous approaches to the study of the relationship between early modern natural philosophy and artisanal knowledge and practices. A seminal and comparatively early attempt is Edgar Zilsel’s thesis on the crucial role of “superior artisans” in early modern Europe (Zilsel 1942: 552–54). Lesley Cormack has proposed a contemporary adaptation of this thesis with the category “mathematical practitioners” (2017a, b).^106^ Also relevant are Smith’s presentation of artisanal epistemology (Smith 2022) and Pamela Long’s use of the concept of a “trading zone” to describe the relationship between artisans and philosophers (Long 2011).^107^ All these categories attribute an active role to artisanal knowledge within the networks of knowledge production. In contrast, Mark Thomas Young advocates a rejection of this “legitimation thesis” regarding artisanal practices, as he terms it (Young 2017: 521–50). Leibniz does not neatly fit into any of these categories. Whereas he expressed much interest and even fascination with artisanal practices, he attributed a lower status to artisanal knowledge, as he claims that it may represent a mystery or a secret to the geometer and philosopher—eventually turning this claim into a call for such knowledge to be included in and subjected to the expanding field of geometry.

To return to the *artiste* (or artisan) as a machine constructor, let us recall that Leibniz described the constructor of the stocking frame as a geometric *ingenio* (genius). Leibniz’s use of the term *ingenio* mirrors an “evolution of early modern ‘ingenuity’ into eighteenth-century ideas of ‘genius,’ and … a social process in which broader ideas of artisanship, craft knowledge, and the workshop would become subsumed by a focus on the virtuosity of the ‘artist’ as ‘individual’” (Murphy 2021: 80). As Marieke M. A. Hendriksen argues, “the common meaning of ‘being a genius at’” was associated with being able to do a certain task “easily and naturally, but not necessarily exceptionally … [and] not demand[ing] singularity, great intellect, or immense creativity.” The meaning of genius, nevertheless, “changed subtly but meaningfully in the course of the eighteenth century. The concept of artistic genius in the sense of a visionary, powerfully possessed artist had slowly started to develop …” (Hendriksen 2021: 139). It is clear that Leibniz attributes to the artisan-constructors of machines not natural and unexceptional abilities but quite the opposite: he considered such constructors to be unique and possessed of individual genius, since “their discoveries are difficult and profound and put forth with much meditation.”^108^ This leads us finally to the discussion concerning what was meant by the idea of geometric knowledge in the seventeenth century.

### 4.2 Conclusion: Mechanical and Geometric Knowledge and the Machines of the Artisans

When examining the great narrative of the “mathematization of nature” during the seventeenth century, Sophie Roux notes (2010) that one sees not only very little agreement that this mathematization was a unified or a unifying project, but also a plurality of types of mathematizations—most clearly observed when one examines the encounters between mathematicians, practical geometers, philosophers, and artisans. While it is clear that even prior to the seventeenth century, practical or mixed mathematics played an essential role in navigation, fortification or masonry—to give only three examples—Leibniz’s emphasis on “extracting” geometrical knowledge from artisanal practices does not necessarily aim to situate such machines in the framework of practical mathematics.^109^ Moreover, following Ofer Gal and Raz Chen-Morris, the changes prompted by the various projects of such mathematization had paradoxical consequences. As Gal and Chen-Morris argue, between the works of Johannes Kepler and Galileo at the turn of the seventeenth century and those of Newton and Huygens during its final decades, the success of applying mathematics to the study of nature undermined the epistemological promise that mathematics initially presented—namely its claim to certainty (Gal & Chen-Morris 2014). Leibniz’s own attempts to point to and extract the more profound, secret, or hidden geometric rules at the base of the machines and artisanal practices he examined may be seen as composing a part of this seventeenth-century “baroque science,” to cite the title of Gal and Chen-Morris’s book. These attempts show that the answer to the question of which objects could be considered geometric changed dramatically during the seventeenth century.

Leibniz’s notion of a secret geometry and his proposed project of expanding geometry do not appear in an intellectual void. On the one hand, the various, at times one-directional mathematical exchanges between natural philosophers and artisans do show that certain mathematical practices were extracted from or applied to “secretive” artisanal practices (for example, mining; see Morel 2023). On the other hand, according to Vincenzo De Risi (2019), Leibniz’s work on transcendental curves was part of his attempt “to surpass the shortcomings of the application of algebra to geometry” (De Risi 2019: 155). The general turn in seventeenth-century mathematics to the increasingly frequent use of algebraic signs and techniques in geometry is well known.^110^ Michael Mahoney has described this turn of geometry—or rather its expansion—as a transition from a geometric to an algebraic mode of thought (Mahoney 1980a: 141–55). This can be traced to François Viète’s notion of *arithmetica speciosa* and was repeated in many formulations and contexts throughout the seventeenth century. In his inaugural lecture at the University of Oxford on October 31, 1649, John Wallis referred to *arithmetica speciosa* in order to propose a project of elevating arithmetic so it would incorporate and ground geometry (Beeley 2011). In 1646, Frans van Schooten published the treatise *De organica conicarum sectionum in plano descriptione tractatus* (On an Organic Description of Conic Sections in the Plane), where he followed his mentor Descartes by presenting techniques for creating curves impossible to achieve algebraically and that could only be drawn by hand (Crippa & Milici 2023). As noted above, in his studies on phoranomics, Jungius considered several curves—classified today as transcendental—to be geometric. In Leibniz’s eyes, however, these attempts were insufficient, since they failed to attend not only to a whole domain of curves but to practices, machines, and natural phenomena. In other words, for Leibniz the notion of secret or hidden knowledge already had a historical or social dimension and even a polemic status.

According to Serfati, when Leibniz first introduced the concept of a secret geometry, he was indeed dealing with transcendent curves, but although these were mechanical in the Cartesian sense, “Leibniz does not even remark upon this” (Serfati 2018: 4). A more explicit polemical dimension to his investigation of the transcendental appears a little later in a 1676 scholium to *Quadraturae circuli arithmeticae pars prima*, where Leibniz explains that a problem is transcendent because “it cannot be in fact reduced to any equation such as Vieta taught us to prepare, nor be built by means of curves such as those introduced by Descartes in geometry” (Serfati 2018: 5). Hence, Leibniz’s project of an expansion of geometry should be seen as a direct criticism of the primary Cartesian distinction between geometric and mechanical curves (Breger 1986; Knobloch 2006; Probst 2012; Blåsjö 2017; Crippa 2014; 2019). Indeed, Descartes might have been the first to propose the distinction between mechanical and geometric curves, though he quite explicitly excluded the former from the realm of geometry. While this has been discussed by various scholars (Crippa 2014; Bos 2001; Blåsjö 2017), and while Leibniz disagreed that these curves could not receive a mathematical treatment, here we explicitly highlight the connection between the secret geometry of transcendental curves and artisanal practices and machines. But for the mechanical to be brought into the geometric, the original distinction between the two must at least be blurred, when the curves found as the basis of secret artisanal machines might act as a mediating link. As Probst (2012) shows, Leibniz’s later typology of curves indeed puts the transcendental and the analytic within the domain of the geometric. However, the mechanical does not completely disappear and is used in 1674 to describe curves that allow only pointwise constructions. This may also explain why when Leibniz brings the mechanical into the realm of such hidden geometry, he examines the principles according to which the artisanal machines operate.

This tension between mechanical and geometric knowledge, principles, and experience helps clarify Leibniz’s concept of a secret or hidden geometry. We saw this with the various concepts Leibniz used: in 1674 and 1675, *geometria arcana* (secret geometry); in 1676, *geometria profundioris*; in 1676, his project of reducing mechanics to geometry, *De arcanis motus*; and in 1686, his *geometria recondita*. These terms are tied to mechanical curves (in the Cartesian sense) but also, and more notably, to new artisanal machines, such as textile machines and timekeeping technologies.

This brings us back to the question of the extraction of knowledge from artisans, as well as to those instances where Leibniz seems to deconstruct the hierarchy between the artisanal-mechanical and the geometric. The association between geometry’s new domains and secret artisanal knowledge is seen when geometry’s existence is justified by the new artisanal machines and technologies it enables, when Huygens’s geometric time-measuring principle is matched by Leibniz’s mechanical principle, when Leibniz expresses his explicit desire to appropriate mechanical knowledge, and when, in the draft of the letter to Oldenburg, he celebrates his new mechanical achievement as unique because it could have been discovered by artisans. This is not a symmetrical trading zone, nor is it a mere legitimation of artisanal knowledge; rather, it is a more distanced or careful interest. Even if this interest shares many characteristics with other thinkers of his time, and is also framed partially by various historical accounts of this relationship in the seventeenth century, Leibniz exhibits a unique, even idiosyncratic, relationship with artisanal knowledge.

## Abbreviations

A: Leibniz’s edited works from Leibniz, G. W. (1923–). Sämtliche Schriften und Briefe. Darmstadt, Leipzig, and Berlin. Deutsche Akademie der Wissenschaften zu Berlin. Abbreviated by the letter ‘A’ followed by one Roman and one Arabic numeral (representing the series and volume number) to refer to the edition of Leibniz’s collected works published in the Akademie der Wissenschaften edition of Leibniz’s works.
LH 38: Leibniz-Handschriften zur Technica, in Gottfried Wilhelm Leibniz Bibliothek. Niedersächsische Landesbibliothek, Hannover.

## Acknowledgements

This research was supported by the Israeli Science Foundation (grant no. 461/21). Yoav Beirach thanks the Technion Department of Humanities and Arts and the Minerva Fellowship of the Minerva Stiftung Gesellschaft fuer die Forschung mbH for supporting this research.

## Footnotes

1 When referring to Leibniz’s edited works, we use the letter A followed by one Roman and one Arabic numeral (representing the series and volume number) to indicate the edition of Leibniz’s collected works published in the Akademie der Wissenschaften edition of Leibniz’s miscellaneous works in Gottfried Wilhelm Leibniz, *Sämtliche Schriften und Briefe*, Berlin-Brandenburgische Akademie der Wissenschaften, Akademie der Wissenschaften zu Göttingen, 1923–. Leibniz’s statement about the need to reveal the secrets and inventions of the Parisian artisans is found in A I1: 416. He notes there that one should “tirer d’eux leurs secrets et inventions.”.

2 Ibid. “… il seroit important, de pécher des ouvriers d’ici le fin et le delicat de leurs secrets.”.

3 Ibid., 417: “Pour moy jay eu loccasion de ne freqventer pas seulement qvantité de bons ouuriers, mais de tirer aussi qvelqve chose deux …”.

4 A IV4: 68: “Bishehr von Theoreticis so den grund zu aller praxi legen, nun komme auff *practica Artium* selbsten, und solche Erfindungen deren Nuzen alsbald erhellet. Habe demnach viel Neue erfindungen, so theils meine inventiones, theils von andern hehrkommen, aber noch wenigen bekand.”

5 For a detailed presentation of the *machina deciphratoria,* see Rescher (2014).

6 Ibid. “Man hat Leute an der Hand, die es in *Machinis Textoriis* weit gebracht, also daß die geblümte figurische bande und Zeuge mit unglaublichen Vortheil und geschwindigkeit zu verfertigen, und weilen dergleichen wenig in Teutschland sondern alles bey frembden gearbeitet wird …”

7 Ibid., 69: “Ich habe eine Invention von Uhrwercken ausgesonnen, welche nicht geschwinder noch langsamer gehen […].”

8 It may seem that the clock artisans are in a better position than other artisans, and they are unique in the sense that they appear only with the machine and do not mechanize an existing, more ancient practice; however, as we will see, also knitting with the stocking frame was regarded as a practice that was entirely new.

9 A VI4: 585–86. Moreover, distinct knowledge can be inadequate or adequate; adequate knowledge can be symbolic or intuitive. Perfect knowledge is intuitive adequate knowledge. For an extensive analysis of these various types of knowledge in Leibniz’s thought, see Esquisabel (2012).

10 A VI4: 586: “Similiter videmus pictores aliosque artifices probe cognoscere, quid recte, quid vitiose factum sit, at judicii sui rationem reddere saepe non posse, et quaerenti dicere, se in re quae displicet, desiderare nescio quid.” See the translation in (Leibniz 1989: 23–24): “Similarly, we see that painters and other artists correctly know what is done properly and what is done poorly, though they are often unable to explain their judgments and reply to questioning by saying that the things that displease them lack an unknown something.”

11 A VI4: 1230: “*confusae cognitionis et distinctae*.”

12 For a more elaborate discussion on Leibniz’s copy of *Logica de notionibus* in the framework of Jungius’s manuscript on weaving *Texturæ Contemplatio*, see Friedman (2023c: 350–56).

13 See also Merchant (2013: 551–99).

14 Long borrows the term from Peter Gallison.

15 On such precarious, unstable, and somewhat imaginary zones, see also Friedman (2023c: esp. 326–29).

16 A I1: 416.

17 *Leibniz-Handschriften zur Technica *(from now on abbreviated as LH 38), fol. 277r.

18 For example, his copy of John Crate’s *Abbildung einer Curiouson Uhr* found in his collection (LH 38, Bl. 378).

19 Letters to Oldenburg and La Roque, March 1675 (A III1: 205–08, 213–14).

20 *Journal des sçavans*, 25 March, 1675, 93–96. See also A III1: 181–201 for earlier versions of the published article.

21 *Journal des Trévoux*, 1718. See Gerland (1906: 131).

22 On the clock metaphor in Leibniz’s writings, see Favaretti Camposampiero (2017); Bobier (2016); Scott (1997). On the clock metaphor in seventeenth-century Europe, see Neumann (2010). Surprisingly, not much has been written on Leibniz and non-metaphoric clocks. Hofmann (1974: 118–21) discusses the different versions of the letter published in the *Journal des sçavans* in 1675 and provides a short description of the watch itself and the new principle underlying it. In his discussion on Leibniz as engineer, Poser (2016: 393) briefly mentions Leibniz’s article in the *Journal des sçavans* and its new principle of isochronism.

23 A III1: 206: “Mitto igitur Tibi, quam vides, descriptionem principii aequalitatis in horologio a me invento futurae, nihil cum isochronismo vibrantium pendulorum aut elateriorum commune habentis, quo tamen uno hactenus omnes usi sunt.”

24 Ibid.

25 LH 38, fol. 277r. Transcription in Gerland (1906: 131): “je publiai un peu après dans les *Journal des sçavans* un autre principe d’égalité, qui n’est pas physique, comme est la supposition de l’égalité des vibrations des pendules, ou des ressorts; mais purement mécanique.” See Sect. 2.2, where Leibniz explicitly notes Huygens’s “spiral vibrating spring,” i.e., the balance spring watch. A few more letters sent from Leibniz to Oldenburg later that year mention his invention and the importance of his new principle, though not in such length. In these letters, Leibniz also replies to some doubts raised by Oldenburg’s acquaintances regarding the functionality of his device.

26 LH 38, fol. 98r. See also Jones (2016: 67).

27 Jones (2016: 7); Morar (2015: 21–22); Nachlass Gottfried Wilhelm Leibniz, Signatur, LBr 489; A1, 17:11.

28 Since the Leibniz–Ollivier relationship has received ample attention in the literature, we will not treat it here; see Jones (2016: 58–80).

29 A III4: 608. “… vostre excellent ouvrage sur les pendules, a esté un des plus grands motifs des progrès que j’ay peutestre faits depuis dans ces sortes de sciences.”.

30 As Leibniz wrote in an unpublished piece on an automaton that synchronized a pendulum clock with a model of planetary motion, *Machina coelestis*, such a device will bring “great use for solving and predicting [astronomical] phenomena” (“magno ad phaenomena solvenda vel praevidenda usu”; Gerland [1906: 139]). Also, in his 1675 article for the *Journal des sçavans*, Leibniz mentions that the main aim of his watch invention was the finding of longitude at sea (A III1: 201). He shared the understanding that clocks were important first and foremost as tools in the arts of astronomy and naval navigation with many other thinkers, including Galileo, Huygens, and Oldenburg.

31 For example, see A VII6: 493, where Leibniz discusses how Huygens perfected Galileo’s work on two distinct levels that give him the priority as the inventor of the pendulum clock: the combinatorial and the geometric. Favaretti Camposampiero (2017) makes a similar claim by relying on a letter Leibniz sent to Thomas Burnett on November 22, 1695 (Leibniz 1978: 169), though he might be overinterpreting the original passage. Note that in this letter Leibniz again stresses the horology artisans’s lack of knowledge, working by trial and error: “Mais ce que des artisans ont fait de cette nature estoît sans le sçavoir et comme en tâtonnant.”

32 *Journal des Trevaux* 1718 (Gerland 1906: 131). “Ce fut environ en 1674 qu’on fit paroitre dans le monde le premier ressort spiral reglant la montre par ses vibrations. Je fus allors à Paris, où Mr. Huguens fit éxécuter par M. Turet, fameux horologer.”

33 Such a view was obviously not unique: Huygens also had convoluted relations with the artisans he worked with. See Leopold (1980).

34 For example, William Oughtred established the “basic arithmetic” of time telling (as opposed to timekeeping) and Huygens established the first mathematical-physical principle of timekeeping based on a geometric presentation (Gerland 1906: 131). Both are given the elevated double status of being makers and inventors at the same time.

35 We will see below that a similar opinion was also expressed regarding German textile artisans.

36 The note is found in LH 38, Bl. 277; Gerland (1906, 132): “J’ai pensé quelquefois à faire executer cette invention, qui promet des nouveaux avantages assez considerables; mais j’ai toujours manqué de l’assistance d’un bon maitre, qui eût la volonté d’y travailler; les ouvriers ordinaires, sur tout en Allemagne, n’ayant point d’envie de s’écarter de leur routine.”.

37 LH 42, 5, fol. 37v–38r. Translation taken from Jones (2016: 74).

38 Cf. Letters to Oldenburg (March 30, 1675, A III 1: 202) and La Roque (mid-March, 1675, A III1: 181); *Journal des sçavans* (1675); *Journal des Treveux* (1718).

39 For reconstructions of a so-called problem of isochronism in early-seventeenth-century Europe, and for more details on Huygens’s work, see Yoder (1988); Buyse (2017); Abattouy (2017); and Ziggelaar (1968, 1973).

40 For a discussion on the reception and influence of *Horologium oscillatorium*, see Mahoney (1980b); Yoder (1988); and Howard (2008). For a discussion on the history and development of the quantified measure of time, see Beirach (2022).

41 See, for example, de La Chapelle (1750: 329), who underlines the difficulty of bending a lath or a blade in the shape of a cycloid for such a pendulum, leading to its abandonment and prompting a return to a circular pendulum (we thank Bertrand Paoloni for pointing out this reference). *The Edinburgh Encyclopaedia* (Brewster 1830: 117) presents a similar account, emphasizing the role of the development of the escapement mechanism.

42 Obviously, also before the introduction of the Cartesian classification of curves, such curves were considered as produced neither by a straightedge and a compass nor as a conic section, but rather by other mechanical means (though not necessarily termed mechanical); however, this may have meant that they were sometimes considered “ungeometrical.” See Bos (2001).

43 LH 38, Bl. 277; *Journal des Trévoux* 1718 (transcription in Gerland 1906: 131). See also Favaretti Camposampiero (2017) for a transcription based on a version published four years earlier: Sully (1714: 115–21). It is important to mention that Leibniz did not claim he discovered the isochronism of springs in general, and explicitly credited Huygens for that. In fact, this idea was already noted by Mersenne in the second book of the *Harmonicorum libri*, in his *Traité de harmonie universelle* (Paris 1636–1637). For discussions on early seventeenth-century theories of vibration and its link to isochronism, see Meli (1996: 38–50); Dostrovsky (1975). Rather, Leibniz’s idea was to repeatedly use only a small portion of the first vibration of the spring. The analogy to the tones of strings, then, should be understood accordingly: it is the fact that the same string with the same tension would produce the same tone that interested him, not that it vibrates isochronally.

44 “Mais il y auroit encor quelque chose à dire de la nature des vibrations des ressorts, dont l’égalité est verifiée par celles des cordes touchées, qui rendent toujours le même ton, quand elles sont également tendües.” LH 38, Bl. 277; *Journal des Trévoux*, 1718 (transcription in Gerland 1906: 131).

45 Leibniz refers here to the spiral balance spring used in watches that Huygens constructed and published in 1675.

46 “Lorsque Mr. Huguens publia son ressort vibrant spiral, je publiai un peu après dans les Journal des Sçavans un autre principe d’égalité, qui n’est pas physique, comme est la supposition de l’égalité des vibrations des pendules, ou des ressorts; mais purement mécanique, consistant dans une parfaite restitution de ce qui doit vibrer, puisqu’alors les vibrations sont égales, parce qu’elles sont justement les memes.” Ibid.: 132.

47 A comprehensive discussion on Leibniz’s notion of a “physical” principle of equality of time is outside the scope of this paper and will be dealt with in a forthcoming paper by Yoav Beirach.

48 “Celuy-là depend d’une observation Physique, au lieu que le mien n’est fondé que sur une reflexion purement mechanique assez aisée” (AIII 1: 194).

49 The article to which the letter refers was published on March 25, 1675.

50 A III1: 206: “Ipse Hugenius, qui nuper, ut nosti, elegantem illam oscillantis elaterii ad horologia applicationem publicavit, plurimum approbavit meam ut novam et pure mechanicam et a nullo experimento physico aut demonstratione geometrica pendentem, ut mirum sit, artifices in eam non incidisse dudum.” This is an unsent early draft of the letter and therefore does not appear in the English edition of Oldenburg’s correspondences. We thank Paolo Rubini for his valuable help in the translation of this passage.

51 AIII 1: 194 (“à laquelle on n’a pas pris garde, faute de l’art des combinaisons, dont l’usage est bien plus general que celuy de l’Algèbre”).

52 For the letter that was eventually sent and its English translation, see Oldenburg (1977: 240). For a discussion on the full content of the letter, as well as comparison between the different versions, see Hofmann (1974: 118–21).

53 A III1: 206: “Plerumque ita evenit, ut uno egregio invento publicato, quale Horologii oscillatorii fuit, aliorum meditationes velut sideratae et in hanc unam defixae habeant aliquid imitationis non facile exuendae: raro animus hac velut praeoccupatione deposita ad diversum quoddam inveniendi principium attollitur. Inventum ipsum quale sit, ex descriptione et figura fudicabis, et si mereri videbitur, Transactionibus Tuis inseres.”

54 See the above discussion on Leibniz’s copy of Jungius’s manuscript *Logica de notionibus*.

55 Draft of a letter to Oldenburg, mid-March 1675 (A III1: 206): “Mihi certe jam a quadriennio nota fuit, cujus testes in Germania Galliaque habeo; sed in controversiam vocat nemo” (To me certainly it [this principle] was already known for four years, of which I have witnesses in Germany and France; but no one calls it into dispute).

56 AIII 1: 194: “Car ayant consideré qu’un ressort estant rebandé au même point se debandera toûjours en même temps; s’il trouve la même liberté de se debander subitement; j’ay inferé qu’on en pourroit employer deux, dont l’un joueroit pendant que le premier mobile de l’horloge debanderoit l’autre. Car ainsi il n’importera pas s’il le rebande plus ou moins viste, pourveu qu’il le rebande avant que l’autre acheve de se rebander, et par consequent l’un délivrant l’autre sur la fin de son mouvement, ce jeu durera toûjours uniformement, et en laissant passer à chaque retour ou periode de ces deux ressorts, une dent d’une certaine roüe entrainée par le mouvement ordinaire, et qui conte les secondes ou autres parties du temps égales aux periodes, nous avons une horloge ou montre telle que nous pourrons desirer.”

57 Note that the article ends with a few quick replies to doubts that were raised against the feasibility of actually using the instrument in sea travel. Here, Leibniz reminds us that the main objective of perfecting timekeeping mechanisms in the manner he suggested in the article is finding longitude at sea.

58 On Leibniz’s work on transcendental curves, see below in Sect. 4.2.

59 On Parisian artisans, see A I1: 415–18; on the stocking frame, see A I1: 417.

60 A VIII2: 127–28: “De artificio puerorum, quo fila digitis implicata educunt. […] De Textoria arte.”

61 A III2: 269, 271, 589.

62 A III3: 293–94: “[i]n re textoria hatt [Crafft] nun miracula vnter handen, es scheinet, daß der gantzen wellt eine newe lection werde vorgeben, ratione usus, ist es in hoc genere der rechte lapis philosophicus.”

63 A III3: 512. In December 1682, however, Crafft reported that he had to stop working with these machines, since the fabrics produced had gone out of fashion and he therefore needed to have a modified machine built (Ibid.: 760).

64 A IV4: 68. Such advantages of the stocking frame and the ribbon loom are again mentioned in 1699 (A III8: 261).

65 A VII6: 487–88.

66 A VII6: 484: “[…] audire subinde fecit querelas de vanitate Geometriae, quibus illa frustra opponat demonstrationes suas, quando non de veritate sed usu quaeritur.”

67 A VII6: 488: “Combinatoria ingenia plus habent felicitatis, minus laboris; simplicia sunt inventa eorum, et paucis verbis tota dicuntur; ut plerumque animadversione potius quam meditatione […].” This distinction is also echoed in Leibniz’s depiction of his new mechanical timekeeping principle as based on the “art of combination” rather than on *algebra *(A III1: 194).

68 A VII6: 487: “et hodieque si quam hoc nomine homines in his studiis versati appellant, ab ingenio illo mathematico laudare quod per longinqua et difficilia, non tentandi arte, aut divinandi felicitate; sed quodam animi vigore, sibi viam facit.”

69 Moreover, in LH 38, one finds two undated sheets describing the stocking frame in Leibniz’s own handwriting, containing also a highly detailed description of how to operate the stocking frame (LH 38, fols. 100r and 101v). For an analysis of Leibniz’s own description of the stocking frame in these folios, see Friedman (2023b: 452–62).

70 A VII6: 493: “applicationem Penduli ad Horologium, quo numerandi abesset labor, et inventionem lineae curvae, cujus evolutione alia rursus curva describeretur a pendulo, quae cordarum circuli proprie tatem haberet, id enim arcus circuli praestare non poterat, unde repetitae diu vibrationes pendulorum haud dubie erroneae.”

71 For a more detailed discussion on the question of how Galileo understood the gap between his actual measurements, his predictions, and the theory meant to explain them, see Ariotti (1972) and Abattouy (2017).

72 A VII6: 493: “Porro alterum praestare Combinatorii, alterum Geometrici ingenii erat utrumque immortali opere Hugenius pulcherrime absolvit.”

73 Ibid.: “Geometriae profundioris.”

74 A IV3: 491: “Sçavoir il faut admettre l’introduction des instrumens, qui abregent le travail, et par le moyen des quels un seul homme peut faire autant que plusieurs.”

75 Ibid.: 492.

76 On such anxieties and the resulting bans and decrees in the Holy Roman Empire during the seventeenth century, see Blaich (1970: 216–25).

77 A IV3: 493. After discussing a similar situation in agriculture and mining, Leibniz claims that the workers, who will allegedly be left unemployed, will be able to find work elsewhere: “Mais on n’a pas eu égard à leur oppositions, et leur apprehension a esté mal fondée, car on les a employés à d’autres choses.”

78 Leibniz uses the term *Spigilicum*, which was used by Crafft when he wrote on the ribbon loom. See Leibniz’s manuscript titled “Observatio Mechanica de Resistentia Frictionis” in: Gerland (1906: 118): “Est textoribus ingeniosoribus instrumentum emblematibus figurandis aptum, quod Spigilicum, ni fallor, vocant, ubi trochleae quaedam suspensae et centro axeque carentes, intusque vacuae tamen circumaguntur. Quin etiam quidem axem rotae horizontalem ex catena suspendit, cui ipse vicem trochleae praeastabat: ita frictio ab incumbente axe orta cessabat.”

79 According to John Spence, Rømer’s “interest in instruments and hydraulics led him to the discovery of the best shape for the teeth of gears to minimize friction, the epicycloid. He sent this finding to Huygens, suggesting that he use it in his clocks” (Spence 2019: 25).

80 A VIII2: 126: “Geometriae est explicare figuras quas natura et ars singulari quadam ratione producit.” The title is written in the center of the right margin on the recto of the folio.

81 Ibid.

82 Ibid. (Struck through in the original.).

83 A VIII2: 126–27: “Geometriae est explicare figuras quas natura et ars singulari quadam ratione producit: […] Geometria Sartorum […] De artificio puerorum, quo fila digitis implicata educunt […] De Textoria arte. De omnis generis telis. Velours etc. De l’instrument des bas de soye.” The “craft of boys” may refer either to the drawloom, where another person—sometimes a young boy (called the ‘drawboy’)—was needed to raise, at each stage, different groups of warp threads, to create the intricate patterns or it may refer simply to string figures. We thank Siegmund Probst for emphasizing this second option.

84 Ibid.: 131. The references are to Gabriel Mouton, *Observationes diametrorum solis et lunae apparentium* (Lyon 1670); Christiaan Huygens, *Horologium oscillatorium* (Paris 1673); Tito Livio Burattini, *Misura universale* (Vilnius 1675).

85 This length indeed comes very close to the meter. Nonetheless, the modern official meter was arrived at by different considerations and using other means, and the entanglement between its history and the history of universalizing the second still needs to be studied.

86 In ca. 1678–79, Leibniz copied Jungius’s manuscript *Texturæ Contemplatio*, repeatedly emphasizing its importance during the years that followed (see Friedman, 2023c). In this manuscript, weaving is considered contemplative, in relation to the domain of contemplative or theoretical geometry, as opposed to practical geometry. Indeed, while in the sixteenth century the distinction between practical geometry and theoretical (or demonstrative) geometry continued to exist, such a distinction began to become less clear-cut; nevertheless, as Angela Axworthy shows, for Nicolo Tartaglia, whose translation of Euclid’s *Elements* was published in 1543, theoretical geometry is considered speculative or contemplative. Additionally, in his *De l’usage de geometrie* (1573), Jacques Peletier discusses the “contemplation” of geometry as well as its “practice and handling,” emphasizing that both possess “speculative and an operative or practical part” (Axworthy, 2022: 11–12 and 32). Jungius and consequently Leibniz may have been aware of these considerations, hence indicating that the “extraction” of theoretical knowledge from (textile) artisanal practices could not be formulated by artisans but only by the mathematician or natural philosopher.

87 A VII3: 555 ff.

88 Citation from Breger (1986: 122).

89 Serfati (2018: 3–5).

90 See A VII3: 555. The first paragraph of *De progressionibus *underlines what geometry cannot take into account: “Progressiones magnitudinum per crementa infinite parva increscentium aut decrescentium figuris quibusdam geometricis continuo quodam motu descriptibilibus repraesentare interest ad mechanicen quoque; nam et vires et motus, sive temporum spatiorumque relationes crementa habent infinite parva: in quo differunt hae progressiones a progressionibus numericis illis, quae exacte per geometriam repraesentari non possunt.”

91 See also (Crippa 2019) on Leibniz’s constructions with strings of various geometrical, non-algebraic curves, as well as (Blåsjö 2017).

92 The copy can be found in the Leibniz-Bibliothek, Hannover, Signatur: Ms. IV 346, entitled “Jungius, Joachim/Phoranomica.”

93 Jungius’s *Phoranomica* was not published until 1691 (Jungius 1691). It should be noted that one does not find any reference to Cartesian classification in this work. Jungius’s work on phoranomics will be thoroughly discussed in a future work by Michael Friedman and Angela Axworthy.

94 See Breger (1986: esp. 127 f.). See also Serfati (2018: 79–80). At this stage, the meaning of the terms “algebraic” and “transcendental” in Leibniz’s writings corresponds to the modern meaning of the terms.

95 See footnote 86.

96 Recall also that Leibniz calls the constructors of the ribbon loom “more *ingenious* weavers” (see Sect. 3.1.2), since they were able to reduce friction.

97 A VII6: 487: “Unde sunt aliqui qui se Geometras esse ignorant ipsi, cum severe tamen et profunde in eo quod intelligent argumento ratiocinentur.”

98 A II1: 418: “… d’autant que je croy me pouvoir satisfaire à present sur les loix de mouvement, par des demonstrations entierement geometriques, sans me servir de suppositions aucunes, ny des principes d’experience …”

99 See A I1: 415–18.

100 He refers to a machine “qvi fait en ouurier des bas et des étoffes de soye, est icy en vogue.”

101 A I1: 416: “tirer d’eux leurs secrets et inventions.”

102 Ibid.: “bien en fer, et il y a des gens qvi ont le secret de jetter le fer dans des formes, et de le limer par apres ce qv’on ne peut pas communement.”

103 Ibid.: 417: “il y a une infinité de curiositez, comme dans l’orfeurerie, emaillerie, verrerie, horlogerie, manufacture du cuir, potterie d’etain, aux qvelles nos estrangers pour la pluspart ne prennent pas garde …”

104 We would also like to emphasize Robert Halleux’s remark (2009: 9): “ce n’étaient pas les mêmes hommes qui écrivaient les livres et qui pratiquaient les arts et métiers.”

105 A IV 3: 492.

106 The notion of “mathematical practitioners” is taken from E. G. R. Taylor’s seminal work *The Mathematical Practitioners of Tudor and Stuart England* (Taylor 1967).

107 Long borrows this notion from Gallison and applies it to early modern networks of knowledge production.

108 A VII6: 488.

109 On the intersecting of practical and theoretical geometry in the sixteenth century, see Axworthy (2022).

110 See, for example, Mahoney (1980a: 141–55); Bos (2001, esp. 211–21, for a conclusion regarding the period before Descartes). Concerning Huygens, see Paoloni (2022: 423–55).
